# Editorial: Emerging trends in real-world pharmacoepidemiology: 2023

**DOI:** 10.3389/fphar.2025.1601477

**Published:** 2025-04-15

**Authors:** Mohammed S. Salahudeen, Tatiane Da Silva Dal Pizzol, Li-Ting Kao, Gregory M. Peterson

**Affiliations:** ^1^ School of Pharmacy and Pharmacology, University of Tasmania, Hobart, TAS, Australia; ^2^ Graduate Program of Epidemiology, School of Medicine, Federal University of Rio Grande do Sul, Porto Alegre, Brazil; ^3^ Department of Production and Control of Medicines, School of Pharmacy, Federal University of Rio Grande do Sul, Porto Alegre, Brazil; ^4^ School of Pharmacy, National Defense Medical Center, Taipei, Taiwan

**Keywords:** pharmacoepidemiology (MeSH), real-world, medication safety, real-world data (RWD), big data, population-level, patient safety, electronic health records

It is our great pleasure to introduce the Research Topic “*Emerging Trends in Real-World Pharmacoepidemiology: 2023*,” published in Frontiers in Pharmacology. This special Research Topic showcases innovative, real-world studies that advance our understanding of medication safety, efficacy, and utilisation across diverse clinical settings and patient populations.

Pharmacoepidemiology continues to evolve rapidly, integrating complex real-world data and sophisticated analytical techniques (Pazzagli et al., 2018; Dimakos and Douros, 2024) designed to assess effectiveness and patterns of medication use (Sabaté and Montané, 2023). The contributions in this Research Topic reflect these advancements and underscore the interdisciplinary and global scope of contemporary pharmacoepidemiologic research.

Several studies in this Research Topic provide valuable insights into the effectiveness and safety of medications in clinical practice. For instance, it was demonstrated that therapeutic drug monitoring of vancomycin blood concentrations was associated with a significantly reduced mortality risk in critically ill patients (Peng et al.). Similarly, a systematic qualitative review elucidated key barriers and facilitators influencing medication self-management in polypharmacy, offering practical strategies for improving adherence and patient outcomes (Jin et al.).

A number of articles in this Research Topic utilised large pharmacovigilance databases, such as the FDA Adverse Event Reporting System (FAERS), to identify new safety signals. For example, novel adverse events were reported for dexmedetomidine (Liu et al.), and strong signals of drug-induced liver injury were associated with certain CDK4/6 inhibitors (She et al.). Studies also highlighted serious adverse reactions, including pulmonary haemorrhage and haemoptysis, associated with bevacizumab regimens (Hu et al.), safety concerns with transthyretin inhibitors (Liu et al.), and potential risks of tumor lysis syndrome with melanoma treatments involving encorafenib and binimetinib (Xia et al.). Further, analysis of real-world safety profiles of cenobamate underscored the importance of pharmacovigilance in clinical decision-making (Chen et al.). Such pharmacovigilance studies emphasise the necessity of heightened clinical awareness and proactive patient monitoring.

Some of the limitations of the pharmacovigilance databases should be acknowledged. In particualr, while FAERS is a valuable tool for hypothesis generation in medication safety research, it is important to recognise critical limitations inherent in such analyses, including voluntary reporting biases, lack of causality assessments, incomplete demographic data, inability to determine the prevalence of adverse reactions, and potential false-positive signals (Sakaeda et al., 2013; Chedid et al., 2018). As recommended by current best practice guidelines, such as the READUS-PV guideline (Fusaroli et al., 2024), robust pharmacovigilance research requires comprehensive methodologies, including systematic reviews, individual case assessments, and sensitivity analyses to validate these findings further.

This Research Topic also includes significant clinical insights, such as identifying patterns of antimicrobial prescribing in surgical units, revealing opportunities for stewardship interventions to improve antibiotic use (Jamaluddin et al.). Further, the retrospective analysis of spontaneous adverse drug reactions in a tertiary hospital illustrated the importance of local pharmacovigilance efforts to enhance patient safety through targeted interventions (Montané et al.).

Additionally, novel findings around dosing strategies emerged, highlighting therapeutic anti-Xa targets for enoxaparin and underscoring sex-based differences in achieving therapeutic anticoagulation (Tinchon et al.). These real-world insights challenge conventional dosing paradigms and suggest the need for more individualised therapeutic approaches.

Collectively, the 11 studies (Chen et al.; Hu et al.; Jamaluddin et al.; Jin et al.; Liu et al.; Liu et al.; Montané et al.; Peng et al.; She et al.; Tinchon et al.; Xia et al.) make significant contributions to the field of pharmacoepidemiology, highlighting both the complexities and potential of real-world medication safety and effectiveness research. The continued integration of real-world data and evidence has been promoted for being better, bigger, brisker, broader, and bolder, positioning pharmacoepidemiology to embrace new challenges and opportunities.

Data-adaptive techniques, such as machine learning, coupled with expert human interpretation, are increasingly essential to fully leverage electronic health records and advance analytical methodologies (Alowais et al., 2023; Javaid et al., 2024; Chaabene et al., 2025). The development of robust and practical methodologies to manage complex and integrated datasets will further advance the field. Building upon its strong foundation, pharmacoepidemiology is well-positioned to advance significantly across several domains and thrive in this exciting era of real-world data and evidence.

We invite readers to explore these insightful articles, hoping they will inspire further research and innovation in pharmacoepidemiology.
